# Case study of the effectiveness of the learning communities program for students in higher diploma in environmental science

**DOI:** 10.1186/2193-1801-3-S1-P2

**Published:** 2014-12-04

**Authors:** Ho-leung Au, Fung-sheung Chung

**Affiliations:** Applied Science Discipline, Hong Kong Institute of Vocational Education, Kragujevac, Hong Kong; Student Development Office, Hong Kong Institute of Vocational Education (Chai Wan), Kragujevac, Hong Kong

## Background

After the education reform started in 2009 in Hong Kong, there were some major effects on the Higher Diploma Programs offered by the Applied Science Discipline, Institute of Vocational Education (IVE). First, the duration of the Higher Diploma Programs was shortened to two years. Second, the science knowledge background of DSE graduates became diversified owing to the change of the curriculum of senior secondary education. Third, more new students of Higher Diploma programs came from secondary school with Chinese Language as the medium of instruction (MOI), but the MOI of the Higher Diploma Programs is in English.

The Applied Science Discipline could foresee that new students might face difficulties in their learning not only for the above-mentioned issues but also because the teaching and learning environment is significantly different at IVE compared with that at secondary school. After discussions with the Student Development Office at IVE (Chai Wan), a Learning Communities Program supported by the Student Development Office was initiated and piloted to help new students of the Higher Diploma in Environmental Science in Autumn 2012. The program was re-run in 2013.

The concept of the learning community was introduced around 1990 with the goal to advance the collective knowledge and in that way to support the growth of individual knowledge [1]. The Learning Communities Program introduced here aimed to help new students of the Higher Diploma in Environmental Science to adapt to the learning environment at IVE, especially for those students who were relatively weak in science knowledge or from secondary school with Chinese Language as the MOI. The objectives of this program were to enable new students to develop learning skills in Applied Science subjects and facilitate their learning process by explaining concepts and encouraging open communication with student mentors from senior level.

## Methods

Both the mentors and mentees were recruited on a voluntary basis. Thirteen mentors and 19 mentees completed the program in 2012, as did 21 mentors and 23 mentees in 2013. The program was evaluated by focus group interviews with both mentors and mentees after program completion. The examination results of the mentees were also obtained for evaluation.

## Results

In 2012, 12 of the 19 mentees passed all six vocational modules in first semester, and only 4 mentees failed in 2 or more modules. In 2013, 11 of the 23 mentees passed all six vocational modules, and only 6 students failed in 2 or more modules. In the focus group interviews, almost all mentees appreciated the mentors’ efforts in the program and agreed that the program was useful to them. Mentors could help them to understand basic concepts so that they could catch up with the curriculum and be more confident when facing examinations. Mentees found that both group tutoring and a collaborative learning model were effective. This finding is in agreement with the concept of the learning community, which helps to build up individual knowledge [2]. Most mentors agreed that they had helped the mentees to participate in the program. The program also provided an opportunity for mentors to apply what they had learnt in the past and consolidate their knowledge. Friendship amongst the mentors and mentees was also built in the program.

## Conclusions

The Learning Communities Programme not only helps new students to catch up with the curriculum of the Higher Diploma Programme, it also facilitates a positive learning environment. This is in agreement with the study result from Chun and Kuh in 2004 [3], who reported a positive link between the learning community and a student’s overall satisfaction with the college and his/her self-reported outcomes.
